# Expanding the actions of Open Government in higher education sector: From web transparency to Open Science

**DOI:** 10.1371/journal.pone.0238801

**Published:** 2020-09-11

**Authors:** Laura Saraite Sariene, Carmen Caba Pérez, Antonio M. López Hernández

**Affiliations:** 1 Almeria University, Almeria, Spain; 2 Granada University, Granada, Spain; Lancaster University, UNITED KINGDOM

## Abstract

Universities have been pressured by governments to change their way of acting and to be more responsible with the requirements of social development to face the challenges of globalization. To this end, universities must use the principles of Open Science, to allow them to be more transparent regarding the dissemination of scientific results. The purpose of this paper is firstly, to determine the progress made in Open Access policies made by the best-ranked universities regarding ARWU. Secondly, to examine influencing factors that enhance the level of openness in researching, in particular, “transparency”, “reputation”, “participation”, “funding”, “foundation” and “size”. The main results show that those private and older universities, best-ranked in terms of excellence researching and those that have been gradually adopting Open Government policies concerning the dissemination of information through institutional web pages and social participation, are the most interested with complying the recommendations established by the authorities of the Open Science projects.

## Introduction

The opening of the data and its reuse is the new vision towards the collaborative Open Government style in the organizations [1–3]. In this sense, universities have been pressured by governments to change their way of acting, to be more responsible with the requirements of social development, and to face the challenges of globalization [4]. Especially, in the context of research and exploitation of their results, being unique to produce, transmit, and disseminate knowledge [5]. As De Blasio [6] notes, digital platforms, institutional repositories, or participatory portals stimulate continuous dialogue and promote knowledge and collaboration processes. Thus, the principles of Open Government allow them to be more open, transparent, efficient, responsible, and collaborative [7].

The concept of Open Government goes back to the 50s [8], although until nowadays there is no consensus on its dimensions [2, 3]. Most of the literature on Open Government coincides with three fundamental pillars established by Obama [9], which are transparency, participation, and collaboration [10, 11].

In addition, Open Government in broad terms is based on collaborative relationships between the institution and its stakeholders [12]. It permits to access the information, to know the actions of the institutions and, therefore, to participate in decision-making [13]. Moreover, facilitates the reuse of the data without any barrier, especially economical one [2, 14]. In this sense, Open Government could be considered the “many to many” information and knowledge channel [15 p. 491].

Under this trend, the opening of the government in universities has become a medium-term key factor for its legitimacy as it provides greater transparency, improves accountability, and satisfies different needs of the society in general and, consequently, has a positive influence in universities’reputation [7].

**Fig 1:**
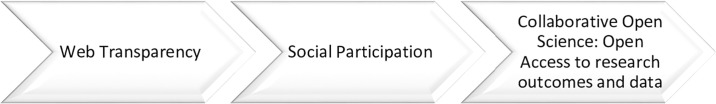
Open Government framework in high education and research institutions.

Thus, the emerged concept of Open Government has been the phenomenon that attracted much interest from researchers in recent years, mainly focusing on web transparency and social participation [2]. However, Open Science and its main extension towards Open Access, framed within Open Government initiatives, is less studied. Therefore, this paper focuses on the focal pillar that supports the principles of Open Government applicable to the high education and research institutions (Fig 1).

As Moedas [16] establishes, science must be open, collaborative, and done with and for society.

According to Gezelter [17], the main objectives of Open Science are transparent methodology, reusability of scientific data, accessibility to scholarly communications, and platforms to facilitate scientific collaboration. In this line, scientific collaboration allows opening the science to all levels of society [18]. Therefore, this openness undoubtedly facilitates progress in the dissemination of knowledge unlimitedly through collaboration on information and digital platforms [19]. Furthermore, it helps to guarantee the quality of the research and the rigorousness of the academic process [20].

Given the previous literature, most of the research deals with theoretical aspects of this way of scholarly communication, so particularly linked to universities [21, 22]. Although today, there is little tendency to share research data in universities [19], Open Access to publications has increasingly positioned as an option for scientists to give visibility to their research [23, 24]. Mainly, the literature at this respect focuses on theoretical aspects of Open Access, explaining the rationale for open initiatives [25]; literature review on the academic, social, and economic impact of Open Access [26], or developing measures of the effect of Open Science collaboration on research and innovation [27].

Others describe indicators to track openness in publications [28]; empirical studies on the collaboration of science and the private sector [29]; data sharing factors [30, 31], or different types of Open Access in various university contexts [32]. Besides, Leiden Ranking has been created based on Open Access indicators [33] or initiatives such as the ranking of Open Access repositories [34] which offers partial information on the share of Open Access availability at the institutional level. The literature on the factors that affect the level of Open Access policies in universities is practically non-existent.

Due to the lack of empirical literature at a global level of Open Access, this paper presents two main objectives. Firstly, to analyze the level of Open Access policies followed by the best-ranked universities. Secondly, to explore the influencing factors of these policies and determine whether the universities, which have achieved better evolution in transparency and participation are getting more progress at Open Access level. The Academic Ranking of World Universities (ARWU) was chosen to gain a global perspective of the possible trends. In particular, the initiatives of Open Access of the top 100 universities were analyzed.

The findings of this study aim to contribute to both the existing literature as well as to identify managerial implications for universities. Therefore, from an academic perspective, this paper seeks to contribute to the research on Open Government. Specifically, to expand the literature focused on Open Science in the higher education sector regarding the level of Open Access policies implementation and its relationship with other dimensions of Open Government. In addition, it can also provide fresh insights about the influencing factors that can lead to greater use of universities’ digital platforms as the channels for improving and facilitating access to scientific information for their different stakeholders.

Moreover, from a practical standpoint, the analysis of the level of Open Access achieved by the top universities in the world can be used as a benchmark by other universities. This study can help university managers to follow the trends of Open Access in the best-ranked universities to reduce barriers to access the literature and lead to a scenario with more computers stage, better connectivity, and technologies. In this sense, this could allow improving and/or developing a more efficient implementation program to advance knowledge.

To achieve the aforementioned objectives, this study is structured in six sections. Following this introduction, the second and third sections provide literature related to the implementation of the Open Access and its influencing factors. The next section details the methodology applied. The fifth section presents the obtained results, and finally, the most relevant conclusions and implications of this research are exposed.

## Open Science initiatives in universities: Open Access

Horizon 2020, the new European Framework for research, and innovation is boosting Open Science to promote scholarly communication [35]. After the publication of the "Open Innovation, Open Science, Open to the World" the European Commission, collaborating with the key stakeholders, has been developing new structures to adopt this new vision of the openness of science [36]. For instance, the “Open Science Policy Platform” [16].

As a consequence of these initiatives, similar policies have been developed and issued in other geographical contexts as “A recommendation on Open Science” [37]; “Open and inclusive collaboration in science: a framework” [38]; “Open science by design” [39], G7 Working Group on Open Science [40] or “Business models for sustainable research data repositories” [41].

The main objectives of Horizon 2020 are to establish mandatory access to scientific publications generated by European funds and to recommend the opening of research databases, which in the end will have to be open by default [36]. In this sense, the European Commission has established a Fair Data expert working group to address the policy and cultural and technological changes facing the opening of science [42]. According to this, Burgelman et al [36] affirm that these policies seek to improve collaboration and engagement of science with society.

Horizon 2020 refers to Open Science as “The transformation, opening up and democratization of science and research through ICT, with the objectives of making science more efficient, transparent and interdisciplinary, of changing the interaction between science and society, and of enabling broader societal impact and innovation”. Consequently, scientific communication can reach anyone with an Internet connection, especially since the social impact is important for developing countries [26].

In addition, OECD [43] highlights the obligation to make publicly funded research accessible through digital formats. In this way, Open Science offers a new approach to the scientific cycle, based on cooperation and dissemination of knowledge using new digital technologies as tools that could boost collaboration [16]. Therefore, this initiative could provide greater accountability, enhance efficiency, and help to face the challenges of general interest [24, 43].

**Fig 2:**
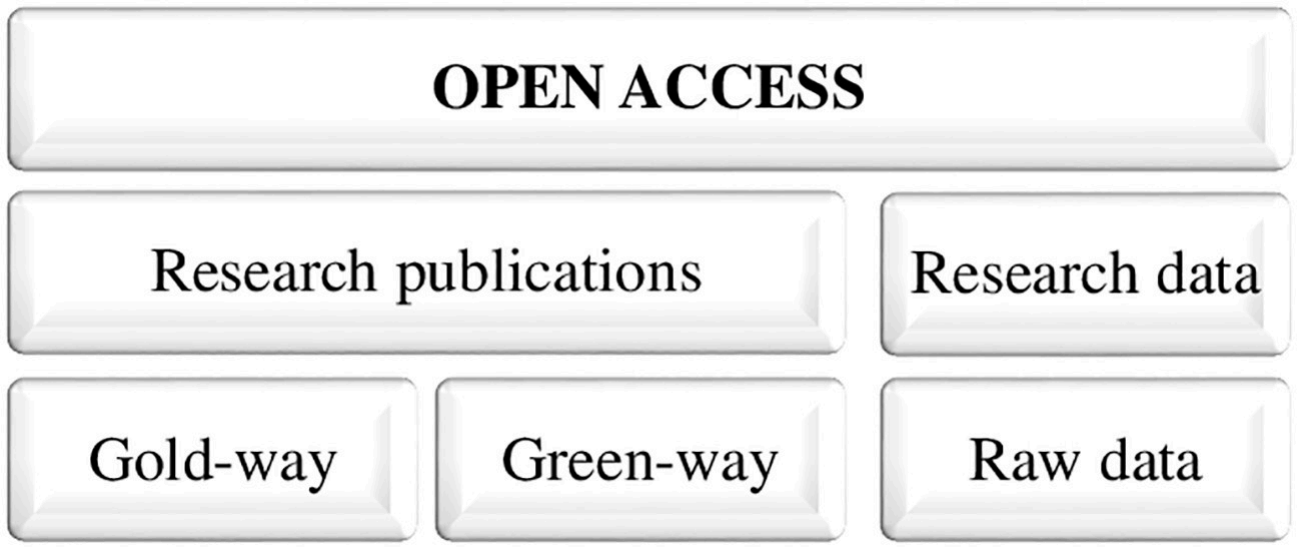
Open Access framework based on the European Commission.

Moreover, Open Science is a broader practice and often referred to as an “umbrella term”, including different aspects of the scientific cycle, highlighting among them Open Research Data and Open Access to publications, on which this study is focusing [18, 22, 38]. In this respect, the European Commission [44] has established the framework (Fig 2) and guidelines on Open Access both to research data and to scientific publications.

In recent years, both governments and funding agencies have strived to consolidate an open research agenda to support Open Access [45]. Especially concerning to publicly funded research [20]. In this sense, different Declarations and Statements have been developed, for instance, the Berlin Declaration [46], the WSIS Declaration [47], the Budapest Declaration [48], or the Public Library of Science [49]. In this respect, Piwowar et al [50] indicate that the US National Institute of Health, the European Commission, the US National Science Foundation, or the Wellcome Trust, among others funding institutions, increasingly make Open Access to the obtained results mandatory. According to the Registry of Open Access Repository Mandates and Policies [51], there are more than one thousand different policies, recommendations, and mandates on Open Access and, in particular, more than eight hundred related to universities and research institutions.

Previous literature highlights that, especially, the universities of Europe and the United States have made an effort to open up the science more than the rest of the world [32]. In this sense, the Association of College and Research Libraries [52] states that the United States Government is taking proactive actions in the same direction as the European Union to adopt measures that require its funding agencies to open scholarly communication. However, for a successful implementation of such policies, adequate infrastructures were necessary, for example, the Open Air platform has been developed, to manage and monitor the European public-funded scientific communication [45].

According to the Budapest Declaration [48], the principles of Open Access defend “free availability of (scholarly) literature on the public Internet, permitting any users to read, download, copy, distribute, print, search, or link to the full texts of these articles, crawl them for indexing, pass them as data to software, or use them for any other lawful purpose, without financial, legal, or technical barriers other than those inseparable from gaining access to the internet itself”. Although it is important to highlight that "free" is for the end-user of the research, since Open Access involves different subtypes such as Gold, Green, Hybrid, Libre, Gratis, or Black [50]. However, this literature review focuses on the Green and Gold types for representing the largest groups of Open Access publications [32] and for having a greater relevance to the main objective of this paper.

The Gold way could be considered when authors submit their research to Open Access journals, which give immediate visibility to the online article. Two important aspects have to be discussed under this modality, the copyright and the article publication costs (APCs). On the one hand, the copyright is generally protected by Creative Commons (CC) licenses, which are applied within the legal framework and help authors to maintain control over their research [53]. These open licenses generally impose few restrictions and offer six different models, being CC BY and CC BY-SA “free license” [43]. In this line, McKiernan et al [54] indicate that retain author rights and control reuse with open licenses, fosters collaboration. Even so, everything under license can inhibit scientific communication [38]. On the other hand, depending on the business model they follow (for profit or not for profit), the APCs are covered either by the authors or are publicly sponsored [43]. Among the largest Open Access journals are the Public Library of Science, Biomed Central, or Springer Open Choice Publishing, for instance [43].

Regarding the Green-way, it refers to the authors’ self-archiving the preprint or postprint versions of their articles. Usually, they provide access to the research through Institutional Repositories or their webpages. Most of the papers published under this modality do not meet the rigorous definition of Open Access, since they imply a period of the embargo as they are first published through traditional channels (journals under subscription fees) and do not extend reuse rights [43, 55]. This goes against the principles of Open Access and confronts the Green-way with the immediacy of the Golden path definitely [56].

In general terms, the previous research agrees that half of the literature is Open Access, where English universities are the most proactive in the implementation of these policies [32, 57]. However, in the last years, university budgets have undergone changes, making it difficult to access all the journals and causing the loss of impact for many of them [16, 58]. Thus, the Golden-way has managed to position itself in front of the Green-way. In this regard, the literature agrees that open articles have a greater impact compared to those that cannot be accessed immediately, the former achieving more citations [50, 58, 59]. Piwowar et al [50] and Peroni et al [59] find that this increase is around 18 percent and between 9–30 percent, respectively. In the same line, McKiernan et al [54] and Wang et al [60] indicate that Open Access articles receive more attention in Social Media. In addition, Abadal et al [61] in their qualitative study find that publishers think that Open Access allows the better dissemination of content, but does not influence its quality.

Furthermore, Yang and Li [62] discuss the lack of peer review in some of the articles of the Golden-way, which increases the lack of confidence, especially in the aspects of plagiarism. Moreover, Dawson and Yang [63] argument that the publications deposited in Institutional Repositories avoid such problems, since they pass rigorous peer review processes, although they present embargo problems to reach greater immediate visibility. However, other studies offer contrary annotations, indicating that Open Access journals are also very exhaustive in their peer reviews [54].

Nevertheless, journals increasingly are giving the author the option to self-archive. In this line, as the recommendations advance towards the official mandate, Open Access Institutional Repositories have become a tool that is settling on the foundations of Open Science [23]. This is confirmed by Piwowar and Vision [64], who found evidence that publications with open databases in repositories get about nine percent more impact than those, which do not reveal their raw data.

Finally, Open Access culture creation is another important aspect of this issue, where employees play a key role [65]. Libraries and librarians are the most important in defending and supporting Open Access policies [25]. Although most libraries do not discuss copyright issues with commercial publishers, leaving it to the authors [63].

## Explanatory factors of academic communication

The theoretical framework for this study is drawn from the dissemination of information and communication management since Open Access emerges as a response to minimize the economic barriers of the traditional system of scholarly communication [66]. This vision being broad, different theories can be considered to explain the dissemination of scientific results from different points of view. Fundamentally, Open Science initiatives seek the informative satisfaction of the organization’s stakeholders, both internal and external. In this sense, among the theories that can best explain the commitment of stakeholders in the websites and digital platforms of an organization are the Theory of Stakeholders [67], the Theory of Dialogic Communication [68] and Legitimacy Theory [69].

Based on these theories and previous literature, the following factors have been selected in order to know the degree of execution of Open Access policies: organizational size [70, 71]; reputation [72, 73]; and age [74]. Other factors to be considered include transparency and public participation [75, 76]. This paper examines the factors most appropriate for its objective, considering the following: “transparency,” “participation,” “reputation,” “funding”, “foundation” and “size.”

According to the stakeholder theory [67], organizations should achieve their objectives with consideration of different stakeholders. In this regard, all entities should inform their stakeholders about the activities carried out [77]. In particular, in the field of universities, after the cases of fraud in recent years, universities following the FOIAs have made an effort at first to access information, that is, transparency. For later, to continue advancing in line with the social demands of the academic world for greater accountability in Open Science [24]. In this respect, the Open Access approach is an efficient way to give diffusion to the scientific results obtained in universities. Cerrillo-i-Martínez [76] states that it is not enough to offer a large quantity of information to satisfy the demand of the stakeholders since the quality of the content and access to it through different mechanisms play an important role. These could be the institutional repositories of the universities that are dedicated to managing Open Access policies. Considering this, the following hypothesis aims to demonstrate the transparency effort to reinforce the level of Open Access in universities:

*H1*: *Achieved transparency positively influences the Open Access level in universities*.

The stakeholder theory points out that long-term organizational outcome is determined by stakeholder relationships [78]. Within the context of universities, academic outcomes are one of the prestige indicators of social interest [72]. Such reputation or prestige is achieved by improving different organizational systems in order to position the university in the different university rankings [79], which measure the quality of research and education [80]. These achievements could affect different groups: students, both current and future, in choosing their studies; employees in the hiring process; and even the process of raising funds or undertaking reforms [81]. Consequently, it would be reasonable to expect that those leading universities would be the most incentivized to use Open Access as mediums to inform their stakeholders of the entity’s excellence in research. Therefore, the following hypothesis is:

*H2*: *Reputation positively affects the Open Access level in universities*.

According to the theory of dialogic communication, Kent and Taylor [68] have developed a framework that explains how it is possible to build and maintain online relationships between an organization and its stakeholders. This dialogic communication theory points out that improving online interactivity creates social relations, increases confidence in the entity, and gives greater satisfaction to the users of these interactions [82]. In this sense, as the new indicators for scientific communication are through Web 2.0 [83] it can be expected that greater participation in social media can influence the higher levels of Open Access in universities. Thus, the following hypothesis is:

*H3*: *Participation positively influences the Open Access level in universities*.

Moreover, Suchman [69] posits that legitimacy is created subjectively as it strongly depends on the perception that the audience has of the organization. Likewise, the author argues that "legitimacy management rests heavily on communication" [69 p. 586]. Therefore, organizations are interested in strategies that can boost the level of participation and collaboration between the firm and the society, using ICTs in order to ensure stakeholders’ comprehensibility and approval of the activities they carry out [84]. At this point, the pressures, in terms of data sharing, that can be received by scientists of public universities from funding agencies can positively influence the attitude towards the dissemination of their scientific results [31]. In this sense, the scientific community increasingly agrees to open publicly funded publications for the interest of stakeholders [85]. Further, the journals are also inciting academics from public and private universities to open both, publications and research data [24, 86]. Thus, universities to lead with this requirement should increase the Open Access policies, in order to gain legitimacy and efficiency. Considering above, the following hypothesis is:

*H4*: *Funding influences the universities’ Open Access level*.

Given the demand for greater legitimacy, efficiency, and transparency [87], older institutions must use the disclosure of information via different digital platforms, not only to improve the visibility of their actions but also, as part of their differentiation strategy [88]. Concerning higher education, Gallego-Álvarez, Rodríguez-Domínguez, and García-Sánchez [89] and Garde-Sánchez, Rodríguez-Bolívar, and López-Hernández [90], consider that organizational age is a relevant factor that should be taken into account when analyzing the access to data of universities. Likewise, Garde-Sánchez et al [74] pointed out that the oldest universities, which have a greater experience in running the organizations than their younger counterparts, are more likely to implement their communication policies better. Consequently, the next hypothesis proposed is the following:

*H5*: *Foundation negatively affects the Open Access level in universities*.

Size is usually related to greater visibility and influence of the organization in society and thus to greater exposure to public scrutiny [91]. Concerning the public sector, Serrano et al [71] point out that the interest of the government to make the information accessible increases according to the size of its population. In the private sector, size is also considered an influencing factor in relation to information disclosure [92]. Conferring to the legitimacy theory it is posited that larger universities would be more interested in offering content with relevant and demanded information in order to improve their reputation, image, and relationships with their stakeholders [74]. Even more, Open Access could be a channel to help developing the correct strategies of Open Government. Thus, it can be assumed that the larger universities have a greater need to share the outcomes of their research. Taking into consideration that larger universities are more likely to adopt open initiatives, the following hypothesis is proposed:

*H6*: *Size positively affects the Open Access level in universities*.

## Materials and methods

### Sample

The sample includes universities of the ARWU’s top 100, commonly known as Shanghai Ranking. Universities are ranked according to several indicators of academic or research performance, including alumni and staff winning Nobel Prizes and Fields Medals, highly cited researchers, papers published in Nature and Science, papers indexed in major citation indices, and the per capita academic performance of an institution. The ARWU is considered one of the most influential and widely used international ranking system of its class because of its solid and transparent methodology [93, 94]. Due to the lack of the necessary data to carry out the explanatory analysis, the final sample consists of 71 universities. The period of the study was September of 2019.

### Analysis of Open Access policies in the best-ranked universities

To achieve the first objective the Open Access initiative in the top universities was analyzed. This analysis is based on Melibea [95], directory, and estimator of institutional Open Access policies of scientific production. This tool allows to compare the content of policy between universities. First, the index related to Open Access policies using indicator estimated by Melibea was elaborated. It is based on the values assigned to a set of indicators (S1 Table), weighted according to their importance in the fulfillment of each aspect analyzed. Second, questions regarding Open Access policies and, according to Melibea, have been sent to those responsible for this issue of the universities that were not available in the directory.

### Explanatory analysis of Open Access

To identify the causal relationship between Open Access policies followed by the top universities and the selected factors six hypotheses were proposed. Assuming linearity in the relationships between the variables studied and, in line with previous literature, multivariate linear regression was used [74, 94]. This is an appropriate technique to identify whether certain independent variables explain a continuous dependent variable [96], particularly if certain organizational factors have explicative power on the level of Open Access policies achieved by universities. The dependent variable “Open Access” (OA) was measured using the index of Open Access developed by Melibea, and the independent variables are shown in Table 1.

**Table 1:** Independent variables.

FACTOR	MEASUREMENT	EXPECTED RELATIONSHIP
Transparency (TRA)	Global Transparency index developed by Saraite-Sariene et al [97] and updated.	**H1+**
Reputation (REP)	The position in Academic Ranking of World Universities (ARWU).	**H2+**
Participation (ENG)	Global Engagement index developed by Saraite-Sariene et al [98] and updated.	**H3+**
Funding (FUND)	Dummy variable, noting 0 in the case of public universities and 1 for private ones [89].	**H4+/-**
Foundation (FOUND)	The foundation date of the university [99].	**H5-**
Size (SIZE)	No. of students [74].	**H6+**

Source: own compilation based on literature review

Taking all of this into consideration, the proposed model for the dependent variable is the following:

$${\text{OA}}_{\text{i}}=\beta_{1}*{\text{TRA}}_{\text{i}}+\beta_{2}*{\text{REP}}_{\text{i}}+\beta_{3}*{\text{ENG}}_{\text{i}}+\beta_{4}*{\text{FUND}}_{\text{i}}+\beta_{5}*{\text{FOUND}}_{\text{i}}+\beta_{6}*{\text{SIZE}}_{\text{i}}+\mu_{\text{i}},$$

where OA is the dependent variable, β the parameters to be estimated, TRA, REP, ENG, FUND, FOUND, and SIZE different independent variables, μ the classic disturbance term; and i refers to each of the universities considered.

## Results and discussion

### Open Access index in the best-ranked universities

The descriptive analysis (Table 2) shows that the level of adoption of Open Access policies is around 47 percent in general terms. Delving further into the analysis of Open Access followed by the universities we can observe that Oxford University is the one that has shown the greater efforts in adopting Open Access policies, followed by Chicago, Illinois at Urbana-Champaign, or Technical Munich universities (S1 Table). Among the universities that did not actively adopt the recommendations of the competent authorities or the implementation of these initiatives is in the development process are Tokyo, Toronto, Peking, or Nagoya universities among others (S1 Table).

**Table 2:** Descriptive statistics.

Variable	N	Min	Max	Mean	SD
Open Access level	71	1	100	47	32,54

Source: own compilation

**Fig 3:**
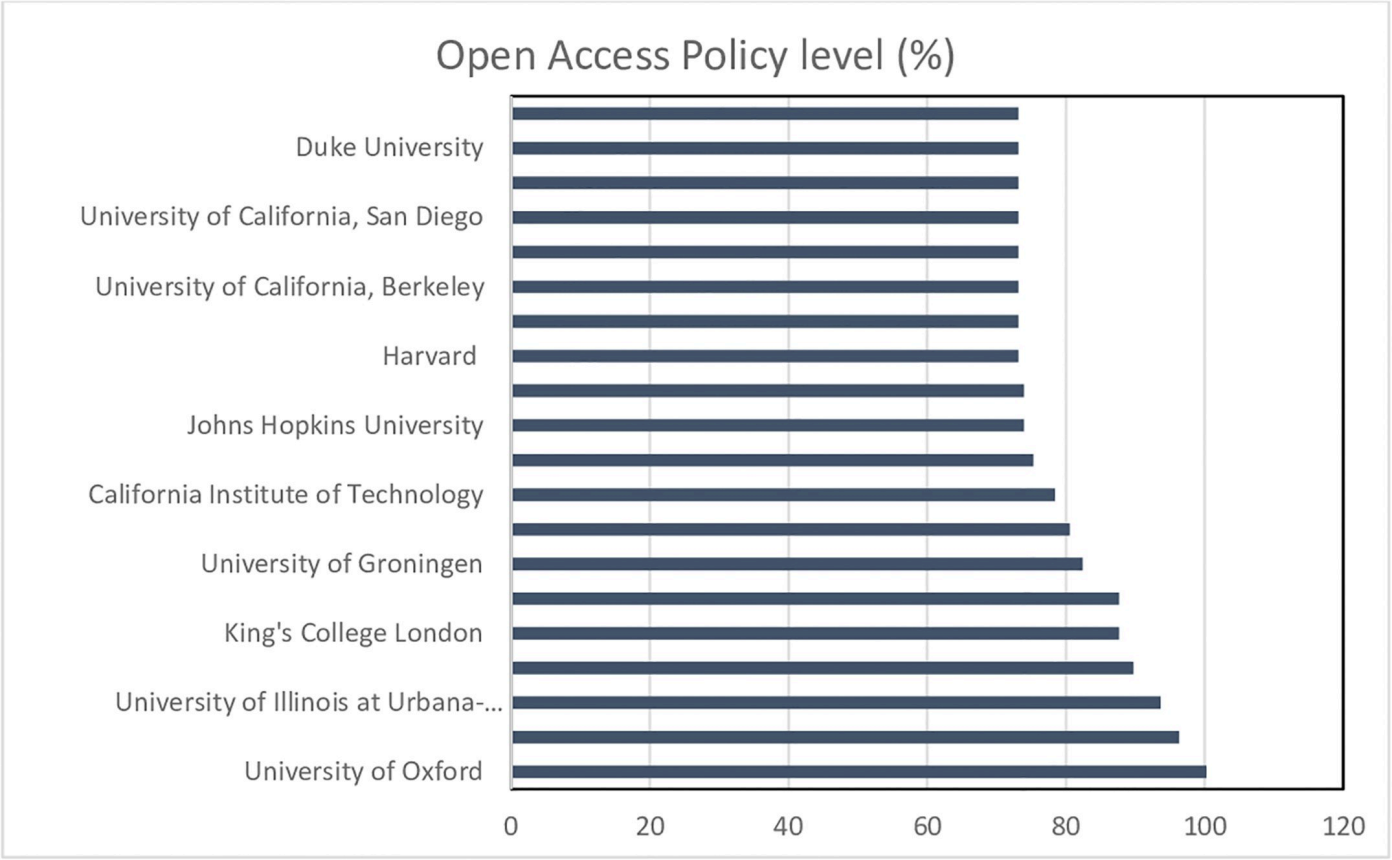
Open Access Policy level of the top 20 universities.

A more graphical view of the best-positioned universities in adoption and monitoring Open Access policies is provided in Fig 3.

Besides, focusing on S1 Table and regarding the “Open Access Policy” only 44 percent of the universities analyzed are applying more rigorously the guidelines proposed by the competent authorities. This is reflected in “Mandatory Compliance” since only 4 percent of the universities allow their staff to be exempt from the deposit and immediate open access without reviewing case by case. Concerning "Deposit Versions", author’s final draft along with the publisher’s versions are the options of more than half of the universities studied (52%), with the “Deposit Deadline” "as soon as possible" being only 8 percent. For the "Embargo Period”, it should be noted that the Green-way and the Golden one get into conflict, 20 percent of universities adapt this problem to the publisher’s stipulations.

Continuing with the “Copyright Reservation”, 40 percent of universities have established that authors maintain copyright, albeit with certain annotations. In addition, the analysis reveals that the deposited material is not used internally, which, in a certain way, confirms that the use and deposit of the raw data of investigations, for example, is in its infancy and many of the institutions of higher education do not mention or are starting to adapt platforms for its reuse. Concerning the “Requirement of Dissertation Deposit”, both mandatory character and recommendation achieve around 30 percent.

Finally, it should be highlighted, that the questions on mandatory compliance, deposit deadlines and embargo period are the issues that are less disclosed; more than half universities do not provide information in this regard.

### Explanatory analysis

The second phase of this study consisted of analyzing the influence of specific factors on the level of Open Access for universities. To this end, a multivariable regression analysis was used. By using Fisher’s critical value (F = 25.52; p<0.01) linearity of the regression was confirmed. After confirming the rest of the null hypotheses of the model (normality, independence, and homoscedasticity), Pearson correlations analysis was conducted. This test revealed significant and positive correlations between the dependent variable (Open Access) and independent variables “transparency”, “reputation”, “participation” and “funding (Table 3). Regarding the independent variables, it is possible to appreciate the relationship between some of them. However, the significant correlation found was lower than 0.8 to provoke problems of multicollinearity in this model [100].

**Table 3:** Bivariate correlation for Open Access.

Variables	**OA**	**TRA**	**REP**	**ENG**	**FUND**	**FOUND**	**SIZE**
**OA**	1						
**TRA**	0.163*	1					
**REP**	0.685***	-0.204**	1				
**ENG**	0.161*	0.331***	-.209**	1			
**FUND**	0.497***	0.166*	.232**	.142	1		
**FOUND**	-0,6	-0.102	.110	-.138	.081	1	
**SIZE**	0,003	0,046	.006	.229**	-.440***	-.011	1

***p<0.01;

**p<0.05;

*p<0.1

Source: own compilation

According to the analysis, the explanatory capacity of the resulting model is 67.4 percent, which was measured using the Adjusted R^2^ (Table 4). As for the proposed hypothesis, five of six were confirmed. In relation to the variable “transparency”, it was statistically significant and relation with “open access” found was positive, confirming the proposed Hypothesis 1 (*β* = 0.177; p<0.05). Thus, the universities which have been making greater efforts in transparency policies over the past few years, are also carrying out the relevant actions for the opening of science. These results are in line with Funamori [24] and OECD [43] who noted that the technological advance information disclosure has increased, affecting the access to scientific publications in the same way. Moreover, to carry out the implementation of Open Science, transparency is a crucial factor that affects accountability in research at all levels in universities [101].

**Table 4:** Regression results.

Hypothesis	Model	Unstandardized Coefficients		Standardized Coefficients	t
		B	Std. Error	Beta	
**H1**	**TRA**	1,226	0,512	0,177	2.393**
**H2**	**REP**	0,096	0,011	0,687	9.120***
**H3**	**ENG**	1,063	0,54	0,154	1.970**
**H4**	**FUND**	4,45	1,102	0,344	4.038***
**H5**	**FOUND**	-0,857	0,478	-0,124	-1.793*
**H6**	**SIZE**	0,746	0,564	0,108	1,323
			R	R Square	Adjusted R Square
			**83.80%**	**70.20%**	**67.40%**

***p<0.01;

**p<0.05;

*p<0.1

Source: own compilation

Following Hypothesis 2, the positive and significant relation between “reputation” and “open access” was found, ratifying the expected relation (*β* = 0.687; p<0.01). Those universities, leading the ARWU, are the most likely to follow Open Access policies. This is in line with Dijkmans, Kerkhof, and Beukeboom [102] who find that reputation is positively related to the online activities of organizations. However, in the university sector, the results are contrary to those obtained by Flórez et al [72], who indicates that reputation does not imply a greater degree in the dissemination of information.

The influence of “participation” was significant and positive, supporting the proposed Hypothesis 3 (*β* = 0.154; p<0.02). This could indicate that the universities achieving the highest levels of participation by stakeholders in social media are the ones that employ the policies with the major requirements regarding the dissemination of scientific results. This outcome can be explained by the appearance of the new indicators (Altmetrics) in the analysis of scientific activity through social media [83]. In the same line, Lampert et al [103] and Serrano et al [104] note that Altmetrics have a potential impact on social engagement in access to scientific information of general interest. In addition, collaboration with citizens also stimulates Open Access, since to achieve greater engagement scientists must give access to the results of research projects to comply with the principles of fair data [105].

Regarding “funding”, it turns to be a significant factor for the model (*β* = 0.344; p<0.01), thus accepting Hypothesis 4. The positive relationship shows that private universities, contrary to what is established in the literature [31, 85] are the ones that most carefully apply the recommendations made by different authorities regarding open access to scientific publications. In turn, the effect found on private funding coincides with the conclusions drawn by Saraite-Sariene et al [97], who find the positive relationship between private funding and information disclosure in the university sector. This may be because private universities, depending on students’ funds, tend to worry more about their reputation, increasing their responsibility for access to all types of information: institutional, academic, research, and in this way strengthen links with their stakeholders.

With respect to “foundation”, significant statistical results were found, confirming Hypothesis 5 (*β* = -0.124; p<0.1). This negative effect is in line with previous research in high education [74] and the corporative sector [106]. In the same vein, these findings support Gallego-Álvarez et al [89], who point out that research groups belonging to the older universities have had more time to consolidate and grow with the consequent need to disseminate more information for different needs.

The results did not support Hypothesis 6 (*β* = 0.108; p>0. 1), thus size does not imply that universities are more prone to Open Science policies. These findings are contrary to the literature on information disclosure [90, 99, 107], where most coincide with the positive effect of size in the dissemination of information in general.

## Conclusions

In recent years, Open Government initiatives have evolved along with ICTs, from the web to social media and digital platforms, which serve for transparency, participation, openness, and collaboration between an organization and its stakeholders. Accordingly, it is necessary to create communities (scientific, governments, private organizations) to improve collaboration both externally and internally of the organization based on technological innovations [108, 109]. The creation of collaboration for open organizations as well as open processes can be carried out through sharing of information, ideas, data, and other resources through digitization with the whole society including, governments, academics, private organizations, and citizens [10, 108]. On this way, Open Access has become one of the main concepts, which is settling on the foundations of Open Science [23].

At this point, different policies have been developed, different pilot projects have been started and various competent authorities [43, 66, 110] have agreed on the requirements of Open Access.

Even so, this study shows that, despite different established policies, until now the level of Open Access policies implementation remains at medium levels in general terms. Likewise, it has been verified the lack of information about many of the elements of the Open Access initiative, as is the case of mandatory compliance, deposit deadlines, or embargo period.

Furthermore, some of the universities are at the beginning stage in the implementation of the recommendations on these open initiatives. Therefore, they do not have managers dedicated to Open Access issues and do not comply with all of the aspects recommended in the official guidelines. Besides, little proactivity is observed in the dissemination of the research data.

Regarding the explanatory analysis and according to Stakeholders, Dialogic Communication and Legitimacy theories five factors should be considered as determinants of the level of Open Access policies followed by universities as part of Open Government strategies: “web transparency”, “reputation”, “social participation”, “funding” and “foundation”.

The level of transparency leads to greater use of digital platforms (for example, Open Access Institutional Repositories) for better openness of research outcomes. This can indicate that the universities which have adapted their web pages to the requirements of access to information have continued along the same lines, advancing and developing institutional repositories, taking into account the requirements and/or recommendations for the transparency of publications.

Likewise, the reputation of the university seems to influence the better adoption of Open Access policies in universities. This may be because the rankings take into account the main indexes of citations, and for a greater impact of the publications greater openness is necessary.

Active communication strategies via social media go in the same direction with Open Access policies. Taking into account the emergence of the new indicators of scientific evaluation through new ICT’s and citizen collaboration in research, scientists tend to use these channels of communication to achieve a greater commitment from society. Moreover, as an accountable response to this, they also tend to share their publications more.

In addition, funding has been a notable driver in the adoption of Open Access policies in universities, with the private universities being those that make the greatest effort regarding the dissemination of their scientific publications. Hence, universities’ behavior is strongly oriented toward meeting the expectations of their funders, including the need to respond to the demand for scientific openness. This helps justifying the funds invested for greater accountability and transparency in research.

Finally, the foundation also influences the best compliance of Open Access policies. These results are in line with the previous literature indicating that most consolidated universities tend to meet the expectations of information demand from their different stakeholders. This is due in part to the fact that the oldest organizations, in order to maintain their competitive advantages, have to adapt their structures and policies to the new technological and social demands.

This study seeks to contribute to both the existing literature and those responsible to manage Open Access policies in the high education field. Therefore, from an academic perspective, the findings aim to provide an overview of Open Science policies in the university sector. Specifically, to expand the scarce literature regarding the level of Open Access policies implementation and its interaction with other dimensions of Open Government initiatives. So, the present paper advance in identifying trends and gaps that should be improved upon for the Open Access policies extension. In addition, it can also provide fresh insights about the influencing factors that can lead to greater use of universities ITC’s as the channels for improving information access, fostering participation, and facilitating access to scientific information for their different stakeholders.

Further, from a practical point of view, the analysis conducted on the level of Open Access in the best-rated universities could serve for other universities as the benchmark practice. This could help to reduce barriers for access to publications and identify the factors that could influence the best adoption of such policies. Knowing the trends in Open Science policies allows improving and/or developing a more efficient implementation program to advance in knowledge. In addition, universities in general, should not delay in adopting the initiatives of Open Science, since it is the best way to deal with legitimacy and accountability with science and with society. Moreover, they should make progress in these policies not only in relation to the dissemination of scientific results but also in the opening of scientific data.

Although this study presents valuable findings, it is not without its limitations, which provides directions for further research. In this regard, the sample size due to the lack of data has been moderately sized. Hence, future research could expand the sample. In addition, the directory for estimating the percentage of Open Access policies does not provide data for all universities. In this sense, once progress is made in the pilot projects of Open Science policies, an analysis of the content could be carried out to prepare an index following the recommendations proposed by different authorities. This analysis should necessarily take into account both the Open Access to the results and the dissemination of the rest of the information of the science cycle, in order to cover the concept of Open Science in its entirety.

As for the explanatory factors, these have been limited and generalized. It would be interesting to expand both internal and external, and in particular, more specific to the higher education sector and top-dawn factors related to the policies in the field of Open Science.

Finally, it has been possible to see the relationship between the three fundamental pillars of Open Government. Therefore, this study could be useful as a basis for future fruitful research on the interrelationships of web transparency, stakeholders ‘engagement, and Open Science in universities. So that, it considers different contexts, by country, by the nature of the funds or see the evolution in the adoption of Open Government.

## Supporting information

S1 Table Open Access index and achieved the Open Access Policy level. (DOCX)

S2 Table Open Access ranking and Arwu positions. (DOCX)
