# Supplementary material for: Expanding the actions of Open Government in higher education sector: From web transparency to Open Science
Source: PLoS One. 2020 Sep 11;15(9):e0238801. doi: 10.1371/journal.pone.0238801 (PMC7485769; doi:10.1371/journal.pone.0238801)
Supplement: S1 Table — (DOCX) [file pone.0238801.s001.docx]

**Table 1. Open Access Index and achieved Open Access Policy level.**

| **OPEN ACCESS POLICY** | **Open Access Policy level** |
| --- | --- |
| Requirement | 44% |
| Recommendation | 22% |
| In the development process | 34% |
| **MANDATORY COMPLIANCE** |  |
| No opt-out of deposit but case-based opt-out of immediate OA | 29% |
| No opt-out of deposit but opt-out of immediate OA | 15% |
| Yes | 4% |
| In the development process | 52% |
| **DEPOSIT VERSIONS** |  |
| Author's final draft and publisher's version | 52% |
| In the development process | 48% |
| **DEPOSIT DEADLINES** |  |
| At the time of acceptance | 17% |
| At the time of publication | 18% |
| As soon as possible | 8% |
| In the development process | 57% |
| **EMBARGO PERIOD** |  |
| 6 months | 11% |
| 12 months | 4% |
| Publisher's stipulation | 20% |
| In the development process | 65% |
| **COPYRIGHT RESERVATION (INSTITUTIONS)** |  |
| Authors retain non-exclusive copyright of self-archive, but with certain rights to the publisher | 40% |
| Authors should retain copyright whenever is possible | 12% |
| No copyright reservation | 6% |
| In the development process | 42% |
| **INTERNAL USE OF DEPOSITED MATERIAL** |  |
| No | 70% |
| Yes | 13% |
| In the development process | 17% |
| **REQUIREMENT OF DISSERTATION DEPOSIT** |  |
| Yes | 32% |
| No | 34% |
| In the development process | 34% |

Source: own compilation according to Melibea.
