# Supplementary material for: Expanding the actions of Open Government in higher education sector: From web transparency to Open Science
Source: PLoS One. 2020 Sep 11;15(9):e0238801. doi: 10.1371/journal.pone.0238801 (PMC7485769; doi:10.1371/journal.pone.0238801)
Supplement: S2 Table — (DOCX) [file pone.0238801.s002.docx]

**Table 2. Open Access Ranking and ARWU positions.**

| **UNIVERSITY** | **Open Access Policy level (%)** | **Ranking ARWU 2018** |  | **Open Access Policy level (%)** | **Ranking ARWU 2018** |
| --- | --- | --- | --- | --- | --- |
| University of Oxford | 100 | 7 | University College London | 50 | 17 |
| University of Chicago | 96 | 10 | Nanyang Technological University | 49 | 70 |
| University of Illinois at Urbana-Champaign | 94 | 39 | Washington University in St. Louis | 46 | 20 |
| Technical University Munich | 90 | 45 | Karolinska Institute | 42 | 42 |
| King's College London | 88 | 53 | University of Bristol | 41 | 59 |
| The University of Western Australia | 88 | 69 | University of Michigan-Ann Arbor | 39 | 27 |
| University of Groningen | 82 | 58 | National University of Singapore | 38 | 66 |
| KU Leuven | 81 | 67 | Swiss Federal Institute of Technology Zurich | 36 | 19 |
| California Institute of Technology | 78 | 9 | Imperial College London | 34 | 24 |
| Stanford | 75 | 2 | University of Pennsylvania | 34 | 16 |
| Johns Hopkins University | 74 | 18 | University of British Columbia | 32 | 41 |
| Swiss Federal Institute of Technology Lausanne | 74 | 64 | Cornell University | 32 | 12 |
| Harvard | 73 | 1 | University of Washington | 28 | 14 |
| Massachusetts Institute of Technology (MIT) | 73 | 4 | Heidelberg University | 26 | 44 |
| University of California, Berkeley | 73 | 5 | Utrecht University | 21 | 49 |
| University of California, Los Angeles | 73 | 11 | University of Toronto | 1 | 23 |
| University of California, San Diego | 73 | 15 | Northwestern University | 1 | 25 |
| University of California, San Francisco | 73 | 21 | University of Wisconsin - Madison | 1 | 28 |
| Duke University | 73 | 26 | Rockefeller University | 1 | 30 |
| University of California, Santa Barbara | 73 | 43 | New York University | 1 | 32 |
| Georgia Institute of Technology | 73 | 62 | The University of Melbourne | 1 | 36 |
| University of Minnesota, Twin Cities | 73 | 35 | University of Paris-Sud (Paris 11) | 1 | 40 |
| Columbia University | 71 | 8 | The University of Texas Southwestern Medical Center at Dallas | 1 | 46 |
| The University of Edinburgh | 70 | 33 | University of Munich | 1 | 50 |
| Erasmus University Rotterdam | 69 | 61 | Peking University | 1 | 54 |
| University of Colorado at Boulder | 68 | 37 | University of Geneva | 1 | 55 |
| University of Zurich | 67 | 51 | Ecole Normale Superieure - Paris | 1 | 57 |
| University of North Carolina at Chapel Hill | 65 | 31 | Technion-Israel Institute of Technology | 1 | 60 |
| The University of Manchester | 64 | 34 | Mayo Medical School | 1 | 63 |
| Princeton University | 63 | 6 | Nagoya University | 1 | 65 |
| University of Cambridge | 60 | 3 | The University of Texas M. D. Anderson Cancer Center | 1 | 71 |
| The University of Queensland | 58 | 52 | The University of Tokyo | 1 | 22 |
| University of Copenhagen | 57 | 29 |  |  |  |
| Monash University | 56 | 68 |  |  |  |
| The University of Texas at Austin | 55 | 38 |  |  |  |
| University of Maryland, College Park | 55 | 48 |  |  |  |
| Ghent University | 55 | 56 |  |  |  |
| Yale University | 52 | 13 |  |  |  |
| Vanderbilt University | 51 | 47 |  |  |  |

Source: own compilation according to ARWU.
